# Mechanische Schutzfixierung – Herausforderungen und Management: Fallvignetten aus der psychiatrischen Intermediate Care Station der Wiener Universitätsklinik

**DOI:** 10.1007/s00739-021-00701-8

**Published:** 2021-02-09

**Authors:** Valentin Popper, Jakob Unterholzner, Lucie Bartova, Alexandra Strnad, Pia Baldinger-Melich, Richard Frey, Gernot Fugger

**Affiliations:** Klinische Abteilung für Allgemeine Psychiatrie, Universitätsklinik für Psychiatrie und Psychotherapie, Währinger Gürtel 18–20, 1090 Wien, Österreich

**Keywords:** Zwang, Rechtliche Aspekte, Klinische Aspekte, Ethische Aspekte, Unfreiwilige Behandlung, Coercion, Legal aspects, Clinical aspects, Ethical aspects, Involuntary treatment

## Abstract

Einschränkungen der Bewegungsfreiheit psychiatrischer Patienten im Sinne einer mechanischen Fixierung sind in Österreich im Rahmen des Unterbringungsgesetzes zur Abwehr von Selbst- und Fremdgefährdung zulässig, sofern deren Anwendung verhältnismäßig ist. Neben rechtlichen Aspekten sind im Rahmen von Bewegungseinschränkungen auf das Krankenbett ethische Aspekte in Zusammenhang mit einem sorgfältigen klinischen Management unentbehrlich. International gibt es Bestrebungen, Zwangsmaßnahmen dieser Art in der Psychiatrie zu reduzieren. Breiter Konsensus besteht darüber, dass deren Anwendung als Ultima-Ratio-Intervention zu sehen ist, die ausschließlich in Situationen eingesetzt werden soll, die nicht durch gelindere Maßnahmen zu bewältigen sind. Die vorgestellten Fallvignetten aus der psychiatrischen Intermediate Care Station der Wiener Universitätsklinik sollen dies verdeutlichen.

## Abstract (en)

Restrictions of the freedom of movement of psychiatric patients in terms of mechanical restraint are regulated within the frame work of the Austrian law on involuntary commitment (“Unterbringungsgesetz”) to prevent endangerment of oneself or of others, provided that the application of these restrictions is proportionate. Apart from legal aspects, ethical considerations related to careful clinical management are crucial in the context of movement restrictions. On an international scale, there are efforts to reduce coercive measures of this kind in psychiatry. There is a broad consensus that their use must be considered as a last resort intervention, to be used exclusively in situations that cannot be dealt with using milder interventions. The case reports presented here from the psychiatric Intermediate Care Ward at the Vienna University Clinic illustrate this.

## Einleitung

Rechtliche Rahmenbedingungen für Beschränkungen der Bewegungsfreiheit psychisch erkrankter Menschen auf psychiatrischen Fachabteilungen sind in Österreich im §33 des Unterbringungsgesetzes (UbG) geregelt. Eine Zulässigkeit für Beschränkungsmaßnahmen nach Art, Umfang und Dauer besteht laut Gesetzestext, sofern diese für die ärztliche Behandlung und Betreuung unerlässlich sind und nicht außer Verhältnis stehen sowie im Einzelfall zur Abwehr von ernstlicher und erheblicher Selbst- oder Fremdgefährdung. Erfasst sind Beschränkungen der Bewegungsfreiheit auf bestimmte räumliche Bereiche. Als Rechtsgrundlage für die Beschränkung auf die Station genügt die Unterbringung an sich, eine weitere Einschränkung auf das Krankenzimmer oder die Eskalation einer Beschränkung auf das eigene Krankenbett im Rahmen von Bettgittern oder einer mechanischen Fixierung (in Österreich als Schutzfixierung bezeichnet) müssen stets ärztlich angeordnet, entsprechend dokumentiert und an die Vertreter der Erkrankten, also jedenfalls die Patientenanwaltschaft und gegebenenfalls die Erwachsenenvertretung, gemeldet werden [1, 2].

Aus ethischer Perspektive repräsentieren die oben genannten Beschränkungsmaßnahmen in der Psychiatrie ein Spannungsfeld zwischen Autonomie und Fürsorge, die zu einem potenziellen Konflikt führen können, wenn Patienten einen Willen äußern, der mit negativen Konsequenzen für die eigene Gesundheit behaftet ist. An dieser Stelle spielt der Begriff Paternalismus aus der Metapher des wohlwollenden Vaters, der für seine Kinder „die beste“ Entscheidung treffen will, eine wesentliche Rolle. Es gibt unterschiedliche Ausprägungen des Paternalismus. Bei Zwangsmaßnahmen wird zwischen starkem und schwachem Paternalismus unterschieden, je nachdem, ob über das Wohl einwilligungsfähiger oder nicht einwilligungsfähiger Patienten entschieden wird. Die Anwendung von starkem Paternalismus erfordert eine Alternativlosigkeit und ist aus moralisch-ethischer Sicht in Ausnahmefällen, also bei nicht einwilligungsfähigen Patienten, gerechtfertigt, um Patienten vor einem Schaden zu beschützen, den sie sich nicht zufügen würden, wären sie nicht psychisch krank. Somit sollte sichergestellt werden, dass unter professionellen und kontrollierten Bedingungen krankheitsbedingtes Leiden verhindert wird [3].

Klinischen Empfehlungen entsprechend sind Schutzmaßnahmen, vor allem die mechanische Schutzfixierung, erst nach erfolglosen verbalen oder nonverbalen Deeskalationsversuchen und stets unter Anwendung effektiver, spannungslösender bzw. sedierender Psychopharmakotherapie einzusetzen. Die mechanische Schutzfixierung wird ausschließlich durch geschultes medizinisches Fachpersonal durchgeführt, wobei die Anwesenheit von zumindest fünf Personen benötigt wird, die jeweils für die Sicherung einer Extremität und des Kopfes der Patienten zuständig sind. Eine engmaschige Überwachung der Vitalparameter, entlastende und empathische Kommunikation, regelmäßige Reevaluierung der Notwendigkeit der eingesetzten Maßnahme (alle 15–30 min) und sorgfältige Dokumentation zählen zu den Standards [4]. Sobald die Schutzfixierung nicht weiter notwendig ist, wird sie stufenweise gelöst [5]. Die Dauer einer Schutzfixierung sollte grundsätzlich so kurz wie möglich gehalten werden, um potenzielle Folgeschäden möglichst zu verhindern. Zu den möglichen körperlichen Schäden zählen primär venöse Thromboembolien [6], welche jedoch durch den Einsatz einer prophylaktischen Antikoagulation verhindert werden können. Insbesondere sollten potenzielle negative psychische Konsequenzen bis hin zur Entwicklung einer posttraumatischen Belastungsstörung (PTBS, engl. posttraumatic stress disorder, PTSD) [7] Beachtung finden.

Die folgenden Falldarstellungen der psychiatrischen Intermediate Care (IMC) Station 04c der Universitätsklinik für Psychiatrie und Psychotherapie (UKPP) in Wien skizzieren vielfältige Situationen, in denen Patienten eine Beschränkung ihrer Bewegungsfreiheit auf das eigene Krankenbett erfuhren.

## Falldarstellung 1

Eine 22-jährige Patientin kollabierte im häuslichen Setting, war nicht mehr ansprechbar und wurde mit der Rettung an die Ambulanz der Universitätsklinik für Notfallmedizin gebracht. Bei bestehender Hypoglykämie (19 mg/dl) und Hypothermie (32,9 °C) wurde die Patientin zuerst auf der Universitätsklinik für Innere Medizin stationär aufgenommen. Aufgrund einer vitalen Gefährdung hatte die Patientin eine Magensonde, einen Harnkatheter und einen zentralvenösen Zugang erhalten und war vorwiegend parenteral ernährt worden. Aufgrund einer seit drei Jahren bestehenden Anorexia nervosa vom restriktiven Typ erfolgten regelmäßige psychiatrische Konsilbesuche. Zum Zeitpunkt der Übernahme auf der psychiatrischen IMC-Station wog die Patientin 26 kg bei einer Körpergröße von 164 cm (Body-Mass-Index, BMI: 9,6 kg/m^2^). Die Patientin konnte weder selbstständig aufstehen noch gehen. Eine aktive Kopfkontrolle war für sie kaum möglich, die Lagerung musste fast komplett durch das Pflegepersonal durchgeführt werden.

Vitale Gefährdung und fehlende Einsicht können den Einsatz von Zwangsmaßnahmen rechtfertigen

Mit engmaschiger physiotherapeutischer Unterstützung wurde sie zuerst passiv und später im Therapieverlauf auch aktiv mobilisiert. Neben den typischen diagnostischen Kriterien der Anorexia nervosa (ICD-10: F50.0) zeigte sich psychopathologisch eine deutlich herabgesetzte kognitive Leistungsfähigkeit mit verlangsamtem Ductus, entsprechend dem reduzierten Allgemein- und Ernährungszustand. Die Patientin konnte sich mit einem sukzessiven Nahrungsaufbau, der fachgerecht zur Vermeidung eines Refeeding-Syndroms [8] sehr sorgsam verlief, nicht identifizieren und war nicht in der Lage, die Tragweite ihrer Erkrankung und die Notwendigkeit der Behandlung zu erfassen, was zu psychomotorischer Unruhe und unzureichender Therapieadhärenz mit vordergründiger Ablehnung jeglicher Psychopharmakotherapie führte. Die Patientin wurde noch am Aufnahmetag nach dem UbG ohne Verlangen untergebracht und bei erheblicher Lebensgefahr gegen ihren Willen über eine Magensonde ernährt und über diese auch medikamentös behandelt. Beidseitige Bettgitter wurden wegen Sturzgefahr in der Nacht eingesetzt. Eine Schutzfixierung war nicht notwendig.

## Falldarstellung 2

Ein 28-jähriger Patient mit bekannter bipolarer Störung kehrte im Frühling 2020 aufgrund sozialer Isolation in Zusammenhang mit der COVID-19-Pandemie von Spanien nach Österreich zurück. Zu diesem Zeitpunkt bestand seine Psychopharmakotherapie aus Lithium. Aripiprazol war aufgrund einer früheren Manie mit psychotischer Symptomatik auch fachärztlich verordnet worden, aber es wurde vom Patienten langfristig nicht akzeptiert und letztlich während der Remission abgesetzt. Im Rahmen der zunehmend manifesten gemischten Episode (ICD-10: F31.6) mit vordergründig dysphorer Stimmungslage, geringer Frustrationstoleranz, ausgeprägter Anspannung mit Agitation und produktiv-psychotischer Symptomatik im Sinne eines religiösen Wahns wurde er initial in einem Versorgungsspital stationär aufgenommen und anschließend an einer Allgemeinstation der Wiener Universitätsklinik für Psychiatrie und Psychotherapie übernommen. Da er sich verbal und körperlich fremdaggressiv mit desorganisiertem Verhalten präsentierte, war bei gegebener Selbst- und Fremdgefährdung eine Unterbringung ohne Verlangen nach dem UbG notwendig. Im Rahmen eines raptusartigen Zustandsbildes zeigten deeskalierende Maßnahmen sowie der Einsatz von spannungslösender und sedierender Psychopharmakotherapie mit Lorazepam intravenös und letztendlich auch Nalbuphin 20 mg.subkutan keine ausreichende Effektivität, sodass der Patient 5‑Punkt schutzfixiert werden musste.

Aufgrund der Notwendigkeit einer komplexen medizinischen Betreuung und des intensiven pflegerischen Aufwandes wurde der Patient zur weiteren fachärztlich-psychiatrischen Betreuung von der psychiatrischen IMC-Station übernommen. Bei weiterhin bestehender ausgeprägter Agitation und konstant fremdaggressivem Verhalten sowie der Ablehnung medizinischer Hilfe und Nahrung war der intermittierende Einsatz mechanischer Schutzfixierung notwendig, welcher von einer sedierenden Psychopharmakotherapie mit Midazolam- und Dexmedetomidin-Perfusor begleitet wurde. Im Rahmen dieser Phase war ein komplettes IMC-Setting notwendig, wobei der Patient konstant monitiert wurde (nichtinvasive Blutdruckmessung, EKG, Pulsoximetrie) und einen zentralvenösen Zugang zur intravenösen Flüssigkeitszufuhr und parenteralen Ernährung erhielt, um einer Aspirationspneumonie vorzubeugen. Aufgrund seiner temporär reduzierten Mobilität erhielt er einen Harnkatheter und eine prophylaktische Antikoagulation mit Enoxaparin. Zusätzlich wurde er intensiv gepflegt und engmaschig physiotherapeutisch betreut. Seine stimmungsstabilisierende und antipsychotische Psychopharmakotherapie wurde unter regelmäßigen Labor- und EKG-Kontrollen adaptiert, wobei Lithium unter engmaschigen Spiegelbestimmungen laufend angepasst wurde und zusätzlich Quetiapin, Risperidon und Valproinsäure verordnet wurden.

Das intensive, multimodale therapeutische Management führte zu einer zunehmenden klinischen Verbesserung, sodass Schutzmaßnahmen schrittweise reduziert und die Psychopharmakotherapie langsam vereinfacht und auf eine perorale (p. o.) Einnahme umgestellt werden konnte. Nach insgesamt etwa drei Wochen war der Patient wieder vollständig mobil und in der Lage, selbstständig zu essen und zu trinken. Er nahm regelmäßig Ergotherapie und ärztlich sowie psychotherapeutisch supportive Gespräche in Anspruch. Abgesehen von kurzen, selbstlimitierenden Phasen dysphorischer Verstimmungen bei etwas verminderter Frustrationstoleranz, war der Patient im Wesentlichen euthym, irritiert bezüglich der vergangenen Wahrnehmungsstörungen, gesprächsbereit, adäquat besorgt, affizierbar, erschöpft, paktfähig und therapieadhärent. Die Unterbringung ohne Verlangen konnte aufgehoben und der Patient auf eine psychiatrische Allgemeinstation verlegt werden. Vor der Entlassung, welche sieben Wochen nach Aufnahme auf eigenen Wunsch erfolgte, wurde mit seinem Einverständnis Risperidon p.o. auf Paliperidon Depot (1 × monatlich intramuskulär) umgestellt. Auch Lithium ist fortlaufend akzeptiert worden.

## Falldarstellung 3

Bei Aufnahme auf der psychiatrischen IMC-Station präsentierte sich eine psychomotorisch extrem unruhige Patientin, die annähernd ohne Unterbrechung lärmte und verlangte, fixiert zu werden, da sie sich sonst verletzen müsse. Im Umgang mit männlichem Fachpersonal zeigte die agitierte Patientin konstant ein hypersexualisiertes Verhalten, häufig von Versuchen begleitet, die männlichen Kollegen zu küssen oder an intimen Stellen zu berühren. Die 39-jährige Patientin hatte im Längsschnitt die Diagnose einer Intelligenzminderung mit deutlicher Verhaltensstörung (ICD-10: F70.1). Sie hatte anamnestisch den überwiegenden Teil ihres Lebens in einer Fixierung verbracht, die seit ihrer frühen Kindheit von ihrer Mutter aufgrund von autoaggressivem Verhalten im häuslichen Umfeld angewendet wurde. In der kleinen gemeinsamen Wohnung ist der junge Mensch durch Beobachtungen, Vernachlässigungen und Übergriffe vielfach traumatisiert worden. Als die Mutter vor einigen Jahren verstarb, wurde die fixierte Patientin neben dem Leichnam aufgefunden.

Die aktuelle stationäre Aufnahme erfolgte als Übernahme aus dem für sie zuständigen Spital, bevor sie in eine speziell organisierte betreute Wohngemeinschaft mit mehrfachen Schutzmöglichkeiten ziehen sollte. Die Patientin selbst gab an, sie sei schon seit jeher fixiert worden, ihre Mutter habe ihr nämlich gesagt, sie müsse sich schlagen, wenn sie nicht fixiert sei. Bei wiederholten Versuchen, die Schutzfixierung unter psychoedukativen und entlastenden Gesprächen zu lösen, konnte dieses Verlangen nach Selbstschädigung tatsächlich beobachtet werden. Zum Beispiel schlug sich die Patientin während der morgendlichen Pflegehandlungen so stark, dass sie sich eine blutende Verletzung an ihrem Ohr zuzog. Die Ohrknorpel waren von früheren Verletzungen beidseits stark vernarbt, weshalb die Patientin auch zeitweise einen Helm trug. Während des 34 Tage dauernden Aufenthaltes wurde einerseits die bestehende Psychopharmakotherapie nach Möglichkeit optimiert, wobei die Patientin Escitalopram, Pregabalin, Topiramat und Zuclopenthixol erhielt. Zudem wurde eine schlaffördernde Medikation mit Prothipendyl und Zolpidem etabliert. Bei Verdacht auf eine Autismus-Spektrum-Störung wurde zusätzlich eine Off-label-Therapie mit Atomoxetin angesetzt. Andererseits fanden intensive verhaltenstherapeutische Interventionen im Sinne eines Tokensystems (Lernen am Erfolg, Belohnungsplan) statt und es erfolgte eine engmaschige ergo- und physiotherapeutische Betreuung. Unter dieser Therapie konnte der Zustand der Patientin etwas gebessert werden, sodass an guten Tagen sogar eine kranielle MRT-Untersuchung ohne Schutzfixierung möglich war. Es kam aber weiterhin fast täglich zu autoaggressiven Handlungen, die auch mit einer 1:1-Betreuung nicht suffizient beherrscht werden konnten und regelmäßige Schutzfixierungen notwendig machten, bis die Patientin wie geplant transferiert wurde.

## Ausblick

Rechtliche, ethische und medizinische Betrachtungsweisen sehen die Beschränkung der Bewegungsfreit von psychisch Erkrankten als „Ultima-Ratio“-Lösung vor. Internationale Vergleiche zeigen jedoch große Unterschiede in Bezug auf Art, Anwendungshäufigkeit und Dauer von Schutzmaßnahmen. Im deutschsprachigen Raum liegt die Häufigkeit der mechanischen Fixierung innerhalb aller psychiatrischen Aufnahmen zwischen 3 % in der Schweiz und 8 % in Deutschland, für Österreich fehlen entsprechende Daten [9]. Generell wird international vermehrt diskutiert, dass eine Reduktion mechanischer Beschränkungen in der Psychiatrie anzustreben ist [10]. In diesem Zusammenhang zeigte sich bisher neben regelmäßigem Training für das psychiatrische Fachpersonal auch eine Erhöhung des Betreuungsschlüssels als wirksam [11]. Berichte über deutliche Rückgänge mechanischer Schutzmaßnahmen nach Modernisierung einer Krankenhausabteilung sind ein Indiz für die Wichtigkeit modifizierbarer Umweltfaktoren als gleich wirksame und weniger invasive Alternativmaßnahmen [12]. Der Idealfall eines personalintensiveren Betreuungsschlüssels, vor allem während der Nachtstunden, würde die Schutzfixierung in einzelnen Fällen ablösen können. Dieser ist jedoch in den meisten Ländern gegenwärtig nicht umsetzbar, was auf mangelnde Ressourcen zurückzuführen ist.

Der aktuelle Konsensus namhafter Experten mit Bezug zur klinischen Praxis lautet, dass ein völliger Verzicht auf Zwangsmaßnahmen in der klinischen Routine nicht umsetzbar ist. Dies ist auch durch unsere Falldarstellungen belegt. Die lebenswichtigen medizinischen Maßnahmen waren bei diesen psychisch sehr schwer kranken Menschen nur durch eine Sicherung möglich, die sogar eine mechanische Fixierung beinhalten musste. Die Anordnung einer Schutzfixierung, die als beträchtlicher Eingriff in die individuelle Freiheit unserer Patienten verstanden wird, setzt detaillierte Kenntnisse über rechtliche Rahmenbedingungen, genauso wie Wissen über psychiatrische und allgemeinmedizinische Standards und eine intensive Auseinandersetzung und Abwägung ethischer Aspekte voraus. Außerdem ist ein engagierter, toleranter, empathischer Zugang essenziell. Gefordert ist das gesamte medizinische Personal, nämlich die im Rahmen des Unterbringungsgesetzes in der Indikationsstellung und Effektuierung verantwortlichen Psychiater sowie die unverzichtbaren Pflegepersonen.

## Fazit für die Praxis

- Eine Schutzfixierung muss immer ärztlich angeordnet werden und ist im Einzelfall zur Abwehr von Selbst- und Fremdgefährdung zulässig, sofern eine Verhältnismäßigkeit besteht.
- Die Anordnung setzt detaillierte Kenntnisse über rechtliche Rahmenbedingungen, Wissen über klinische Standards, eine intensive Auseinandersetzung und Abwägung ethischer Aspekte sowie einen empathischen Zugang voraus.
- Ist eine Schutzfixierung nach verbalen/nonverbalen Deeskalationsversuchen oder dem Einsatz effektiver Psychopharmakotherapie unvermeidbar, muss diese durch geschultes medizinisches Fachpersonal durchgeführt, unter Monitorbedingungen regelmäßig reevaluiert und frühestmöglich schrittweise beendet werden, um potenzielle psychische und physische Folgeschäden zu vermeiden.
- Der gegenwärtige internationale Konsensus lautet, dass Fixierungen möglichst vermieden werden sollen, ein völliger Verzicht in der klinischen Routine aber nicht umsetzbar ist.
